# Effectiveness of nanomaterials and their counterparts in improving rice growth and yield under arsenic contamination

**DOI:** 10.3389/fpls.2024.1338530

**Published:** 2024-05-28

**Authors:** Xiufen Li, Xiaoxuan Wang, Xingmao Ma, Wenjie Sun, Kun Chen, Fugen Dou

**Affiliations:** ^1^ Department of Plant and Environmental Sciences, New Mexico State University, Las Cruces, NM, United States; ^2^ Texas A&M AgriLife Research and Extension Center at Beaumont, Texas A&M University System, Beaumont, TX, United States; ^3^ Zachry Department of Civil and Environmental Engineering, Texas A&M University, College Station, TX, United States; ^4^ Department of Atmospheric and Hydrologic Sciences, St. Cloud State University, St. Cloud, MN, United States; ^5^ Department of Statistics, University of Connecticut, Storrs, CT, United States

**Keywords:** rice, arsenic, nanoparticle, nanofertilizer, zinc, copper, silicon, yield

## Abstract

Arsenic (As) pollution in rice (*Oryza sativa* L.), a staple food for over 3.5 billion people, is a global problem. Mixed effects of Zn, Cu, and Si amendments on plant growth and yield, including in the presence of As pollution have been reported in previous studies. To better investigate the effectiveness of these amendments on rice growth, yield, and As accumulation, we conducted a rice greenhouse experiment with 11 treatments, including control pots with and without As contamination and pots with amendments of ZnO, CuO, and SiO_2_ nanoparticles (ZnO NPs, CuO NPs, and SiO_2_ NPs), their ionic counterparts (ZnSO_4_, CuSO_4_, and Na_2_SiO_3_), and bulk particles (ZnO BPs, CuO BPs, and SiO_2_ BPs). Compared with the background soil, the treatment of adding As decreased rice plant height, panicle number, and grain yield by 16.5%, 50%, and 85.7%, respectively, but significantly increased the As accumulation in milled rice grains by 3.2 times. Under As contamination, the application of Zn amendments increased rice grain yield by 4.6–7.3 times; among the three Zn amendments, ZnSO_4_ performed best by fully recovering grain yield to the background level and significantly reducing grain As^III^/total As ratio by 46.9%. Under As contamination, the application of Cu amendments increased grain yield by 3.8–5.6 times; all three Cu amendments significantly reduced grain As^III^/total As ratio by 20.2–65.6%. The results reveal that Zn and Cu amendments could promote rice yield and prevent As accumulation in rice grains under As contamination. Despite the observed reduction in As toxicity by the tested NPs, they do not offer more advantages over their ionic counterparts and bulk particles in promoting rice growth under As contamination. Future field research using a broader range of rice varieties, investigating various As concentrations, and encompassing diverse climate conditions will be necessary to validate our findings in achieving more extensive understanding of effective management of arsenic contaminated rice field.

## 1 Introduction

Rice (*Oryza sativa* L.) is a staple food for more than 3.5 billion people globally, therefore, any potential health risk associated with heavy metal contaminated rice should be considered with care. Arsenic (As), classified as a *class I* human carcinogen by the International Agency of Research on Cancer (IARC), is among the most prevalent heavy metals in rice paddies and poses a global menace due to its contamination in soil, underground aquifer, and irrigation water (Farooq et al., 2016).

Rice is mostly cultivated under flooded conditions, resulting in anaerobic conditions that promote the accumulation of several-fold higher arsenic in rice than in other cereals (Shikawa et al., 2019; Upadhyay et al., 2019, 2020). In agricultural fields, the average As concentration from the use of arsenic-comprising pesticides and defoliants ranges widely from 5–2,553 mg kg^–1^ (Anawar et al., 2018). Even though nearly all As compounds accumulated from food are toxic to humans and animals, inorganic arsenic is of particular concern and is drawing more attention. Among inorganic As, arsenite (As^III^) represents about 70% of total As in U.S. rice samples (Kim et al., 2013). In flooded rice fields, arsenite (As^III^) and/or dimethylarsinic acid (DMA) are typically the most abundant forms, with low to negligible levels of arsenate (As^V^) and monomethylarsonic acid (MMA) (Meharg and Jardine, 2003). In addition to causing serious food safety concerns, As also negatively affects rice yield. Therefore, urgent solutions are needed to address concerns caused by arsenic pollution and toxicity, including selection of appropriate fertilizer amendments.

Zinc (Zn), copper (Cu), and silicon (Si) are essential micronutrients for plants. A suitable amount of Cu fertilization was shown to increase rice yield as well as N use efficiency; however, excessive Cu could cause toxicity to rice plants (Xu et al., 2006). Many studies have demonstrated that micronutrients packed in nanoparticles (NPs), such as zinc oxide (ZnO NPs), copper oxide (CuO NPs), and silica (SiO_2_ NPs), have a positive effect on plant growth under biotic and abiotic stresses (Liu et al., 2018; Cui et al., 2020; Yan et al., 2021). For instance, under 2 mg L^-1^ As stress, Yan et al. (2021) reported that ZnO NPs at 100 mg L^-1^ decreased As concentrations in rice shoots and roots by 40.7% and 31.6%, respectively, compared to the no NPs amendment control. Sharifan and Ma (2021) compared the foliar application of ZnO NPs and Zn^2+^ on As accumulation in rice and reported that ZnO NPs decreased As accumulation in rice shoots by 28% less while Zn^2+^ decreased As accumulation in rice shoots by 15%. However, it is not yet fully understood whether and to what degree micronutrients packed in NPs can enhance rice growth and yield relative to their conventional ionic and bulk particles, especially under arsenic contamination.

In previous studies, we have reported that nanoparticle amendments and their ionic and bulk counterparts have remarkable impacts on soil pH, redox potential (Eh), soil organic carbon (SOC), cation exchange capacity (CEC), plant available arsenic, and iron plaque in soils at different growth stages of rice (Wang et al., 2021; Wang et al., 2022a, 2022b). In an effort to address the effectiveness and risks of NPs to food safety, our current research aimed to investigate the impact of three agriculturally important nanoparticles including zinc oxide (ZnO), copper oxide (CuO), and silicon dioxide (SiO_2_) nanoparticles (NPs), their ionic counterparts, and bulk particles on rice growth, grain yield, and arsenic accumulation under arsenic contamination.

## 2 Materials and methods

### 2.1 Background soil properties and chemicals

Soil (0–15 cm) was collected from a rice field in Eagle Lake research station, TX (29°38’23”N, 96°20’51” W) on March 3, 2020. Soil was air-dried and passed a 2-mm sieve for use in the greenhouse experiment. The background soil properties are shown in **Table 1**. Briefly, the soil is a Hockley silt loam (fine, smectitic, hyperthermic Typic Albaqualfs) with 19% silt and 15% clay. The background soil contains 1.36 mg kg^-1^ of As, 0.4 mg kg^-1^ bioavailable Cu, and 0.6 mg kg^-1^ dethylenetriaminepentaacetic acid (DTPA)-extractable Zn.

**Table 1:** Background soil properties.

	pH	EC(μS/cm)	WHC (%)	Total C	Total N	NH_4_^+^	NO_3_^-^	P	As	Zn	Cu	Ca	Mg	S	Na
				———————————————— mg kg^-1^ —————————————											
Background Soil	5.9	122	44	8520	818	7.6	45	33	1.36	0.6	0.4	1408	263	11	32

Soil was collected from a research field at Texas A&M AgriLife Research Center at Eagle Lake, TX (29°38’23”N, 96°20’51”W) in 2020, air-dried, and passed 2-mm sieve for chemical analysis. EC, electrical conductivity; WHC, field water holding capacity.

Three metallic oxide nanoparticles (ZnO NPs, CuO NPs, and SiO_2_ NPs) included in this study were obtained from US Research Nanomaterials Inc. (Houston, TX, USA). The primary sizes of ZnO NPs (15–137 nm), CuO NPs (9–22 nm), and SiO_2_ NPs (20–30 nm) were determined using a Tecnai G2 F20 transmission electron microscope as described in Wang et al. (2021). Certified reagent-grade bulk particles, including ZnO BPs, CuO BPs, and SiO_2_ BPs, were obtained from Alfa Aesar (Haverhill, MA, USA). ACS reagent-grade ZnSO_4_·7H_2_O and CuSO_4_·5H_2_O were obtained from Acros Organics Bvba (Geel, Belgium), and certified Na_2_SiO_2_ was purchased from Fisher Chemical (Hampton, NH, USA). NaAsO_2_ was obtained from Sigma Aldrich (St. Louis, MO, USA). Detailed characterization of these nanoparticles and bulk particles has been reported in our previous publications (Wang et al., 2021; Wang et al., 2022a, 2022b).

### 2.2 Experimental design, greenhouse management, and plant and soil sampling

The greenhouse experiment was conducted in a randomized complete block design (RCBD) with four replicates. A total of 44 pots were included in this study (11 treatments × 4 replicates with rice plants). The 11 treatments include one negative control without any amendments (Background), one positive control with 5 mg kg^-1^ of freshly introduced arsenic (As Control), three treatments with 5 mg kg^-1^ of As and 100 mg kg^-1^ zinc amendment (ZnO NPs, Zn^2+^, or ZnO BPs), three treatments with 5 mg kg^-1^ of As and 100 mg kg^-1^ copper amendment (CuO NPs, Cu^2+^, or CuO BPs), and three treatments with 5 mg kg^-1^ of As and 500 mg kg^-1^ silicon amendment (SiO_2_ NPs, SiO_3_^2–^, or SiO_2_ BPs).

Before planting, 4.5 kg dry soil was added to each pot (CN-NCE-0600, Elite Nursery, The HC Companies, Inc.) with a top diameter of 22.86 cm, a height of 21.59 cm, and a volume of 6 L. Arsenic, nanoparticles, ionic counterparts, and bulk particles were mixed with soil in each pot accordingly. Briefly, 5 mg kg^-1^ of As was added to each pot except for the background control pots to keep the concentration of As in line with the average As concentration in the U.S. soils according to the United State Geological Survey soil sampling and report (Punshon et al., 2017). Concentrations of nutrient amendments (100 mg kg^-1^ Zn, 100 mg kg^-1^ Cu, and 500 mg kg^-1^ Si) were selected to avoid phytotoxicity while potentially modifying soil properties according to Liu et al. (2018) and Wang et al. (2021). After soil preparation, 1,386 ml rainwater (non-saline or arsenic-contaminated) was added to each pot to reach 70% water holding capacity (WHC). All pots were placed in three cement trays (1.5 m in length × 2 m in width × 30 cm in height) with each tray as a block. All plots were placed in a greenhouse for 48 hours before planting to ensure homogenization. Rainwater was collected and stored in large containers equipped in the greenhouse for irrigation to simulate the field conditions in rice production in practice. The reuse of rainwater is a common practice in rice production in Texas as a measure to mitigate the water shortage caused by global climate change.

A high-yielding long-grain hybrid variety, XP753 (RiceTec Inc., Alvin, TX, USA) was used in this study. Rice seeds were pre-germinated at 30°C for 36 h for use. At planting [May 21, 2020; 0 day after planting (DAP)], seven pre-geminated seeds were drill-seeded to 1–1.5 cm in each of the 44 pots using tweezers. For the first 28 days after planting (0–27 DAP), 100 ml rainwater was added to each pot every other day to keep moisture of the soil surface. Rice seedlings in each pot were monitored to determine the optimum time to thin the seedlings in each pot. On 22 DAP, seedlings were thinned to four seedlings in each pot.

On 7 DAP, a depth of 10 cm water was added to the cement trays to maintain the temperature of each pot. The water levels in the trays were maintained throughout the experiment until the harvest. On 28 DAP, a depth of 9 cm water was added to each pot (permanent flooding). The water level within each pot was maintained throughout the experiment until the rice harvest. Nitrogen fertilizer (Urea, 46–0-0) were applied at 250 kg N ha^-1^ in two split, with one half (0.77 g/pot, 125 kg N ha^-1^) applied at permanent flooding on 28 DAP, and the other half applied on 53 DAP, 5 days after the maximum tillering stage. Phosphate fertilizer (Super Triple Phosphate, 0–45-0) and potassium fertilizer (Potassium Chloride, 0–0-60) were applied at 40 kg P ha^-1^ and 60 kg K ha^-1^, respectively, on 60 DAP. All pots were hand-weeded throughout the experiments. Rice plants were hand-harvested on September 2, 2020 (104 DAP).

### 2.3 Determinations of agronomic traits

At the maximum tillering stage on 47 DAP, rice tillers were counted, and the leaf chlorophyll index (or leaf greenness) was determined using Minolta Chlorophyll meter SPAD-502 (Minolta Co., Ltd., Japan). Panicles were counted manually for each plant of each pot at the heading stage on 70 DAP and at harvest on 104 DAP, respectively. The fresh weight of panicles at harvest was determined using a Cole-Parmer symmetry EC-series balance (Cole-Parmer Instrument Company, LLC). Rice panicles were separated into two portions, one portion was air-dried in the greenhouse for 7 days until a constant weight for the determination of agronomic traits, and the other portion was oven-dried at 70°C for 7 days for As concentration measurement. The dry weight of the panicles of each plant was determined using a balance. The grain and non-grain portions were separated from the panicles and weighed separately. Rice panicles were threshed manually and cleaned to separate the grain and non-grain portions. After manually removing the non-grain portion, the weights of filled grains and unfilled grains were determined using a balance. A hundred filled grains and a hundred unfilled grains were randomly selected and weighed, separately. If the total number of grains of each plant is less than 100, then the actual number of grains were recorded. This value was used to calculate 1000-filled grain weight and 1000-unfilled grain weight.

### 2.4 Determination of As accumulations in rice milled grains and grain hulls

The total As concentration and speciation in the rice hulls and milled grains were determined with an inductively coupled plasma-mass spectrometry (ICP-MS 7500cs, Agilent Technologies, Santa Clara, CA, USA) after acid digestion following Wang et al. (2022b). Rice grains were oven dried at 70°C for 7 days and milled using a rice mill (Industrias Machina Zaccaria) for 74 seconds to separate the hulls and grains. A 5 mL of 70% nitric acid solution was added to 1.0 g milled grains and 1.0 g grain hulls separately overnight. The mixture was heated at 95°C for 4 hours using a DigiPREP MS hot block digester (SCP science, Clark Graham, Canada). The solution was digested with 3 mL of 30% (w/v) H_2_O_2_, heated at 95°C for another 2 hours, and determined with ICP-MS following Wang et al. (2022b).

### 2.5 Statistical analysis

Analysis of variance (ANOVA) was performed using R 4.2.1 (http://www.r-project.org/) to evaluate the effect of three metallic oxide nanoparticles (NPs), their ionic counterparts, and bulk particles (BPs) on rice growth, grain yield, yield components, grain quality, and As accumulation. *Post hoc* tests were conducted using Tukey’s HSD at a 5% level of significance. Pearson correlation coefficient and p-value were used to examine the relationship between tested soil properties, agronomic traits, and As accumulation in rice.

## 3 Results

### 3.1 Rice plant growth

**Figure 1:**
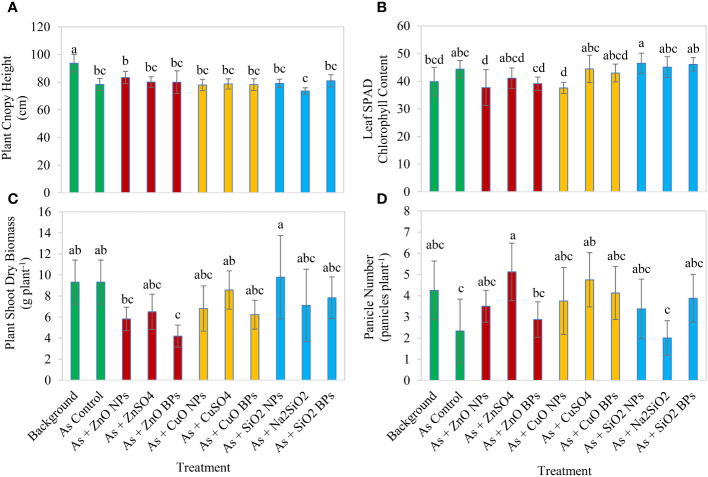
Effect of three metallic oxide nanoparticles (NPs), ionic counterparts, and bulk particles (BPs) on rice growth, including plant canopy height, leaf SPAD chlorophyll content at 82 days after planting **(B)**, shoot dry biomass **(C)**, and panicle number **(D)**. Eleven treatments included: one background soil without amendment; one background soil with arsenic addition only (As control); background soil with As addition and amendment of a metallic oxide nanoparticle (ZnO NPs, CuO NPs, or SiO_2_ NPs); background soil with As addition and amendment of a ionic counterpart (ZnSO_4_, CuSO_4_, or Na_2_SiO_2_); and background soil with As addition and amendment of a bulk particle (ZnO BPs, CuO BPs, or SiO_2_ BPs). Letters on the bars denote statistical significance.

Rice plant canopy height in the background soil with no amendment was 93.8 cm, whereas in the control soil adding As only, it significantly decreased to only 78.4 cm (*p* ≤ 0.05) (**Figure 1A**). Under arsenic stress, application of nanoparticles, ionic counterparts, or bulk particles at the tested concentrations did not lead to significant improvements in plant height. There was no significant difference in plant height among the micronutrients (**Figure 1A**).

The presence of arsenic contamination at a level of 5 mg kg^-1^ did not significantly alter leaf chlorophyll SPAD index 82 DAP compared with the background soil (**Figure 1B**). Under arsenic stress, application of ZnO NPs and CuO NPs significantly (*p* ≤ 0.05) reduced the chlorophyll SPAD index of rice leaf, whereas all other amendments did not significantly impact chlorophyll SPAD index when compared to the rice plants in the As control treatment. In contrast, adding Si-containing amendments promoted leaf chlorophyll content, particularly for the SiO_2_ NPs which significantly increased leaf chlorophyll content by 1.1–1.2 times compared with the background treatment. There was no significant difference in chlorophyll SPAD index among the nanoparticle, ionic counterpart, and bulk particle form for all three micronutrients.

Arsenic contamination at 5 mg kg^-1^ did not significantly affect rice shoot dry biomass, which was 9.3 g plant^-1^ in both background soil and As control soil (**Figure 1C**). When compared with As control soil, only ZnO BPs amendment significantly (p ≤ 0.05) reduced plant dry biomass; by contrast, other amendment did not significantly affect plant shoot dry biomass. Compared with ZnO NPs and CuO NPs, SiO_2_ NPs increased plant biomass production.

The number of panicles per plant was 4 in background soil but was only 2 in As control soil; however, the difference was not significant (**Figure 1D**). Compared to As control soil, ZnSO_4_ and CuSO_4_ significantly increased the panicles number by 2.2 and 2.1 times, respectively. Numerically, all amendments promoted panicle production except Na_2_SiO_2_.

### 3.2 Rice grain yield and yield component

**Figure 2:**
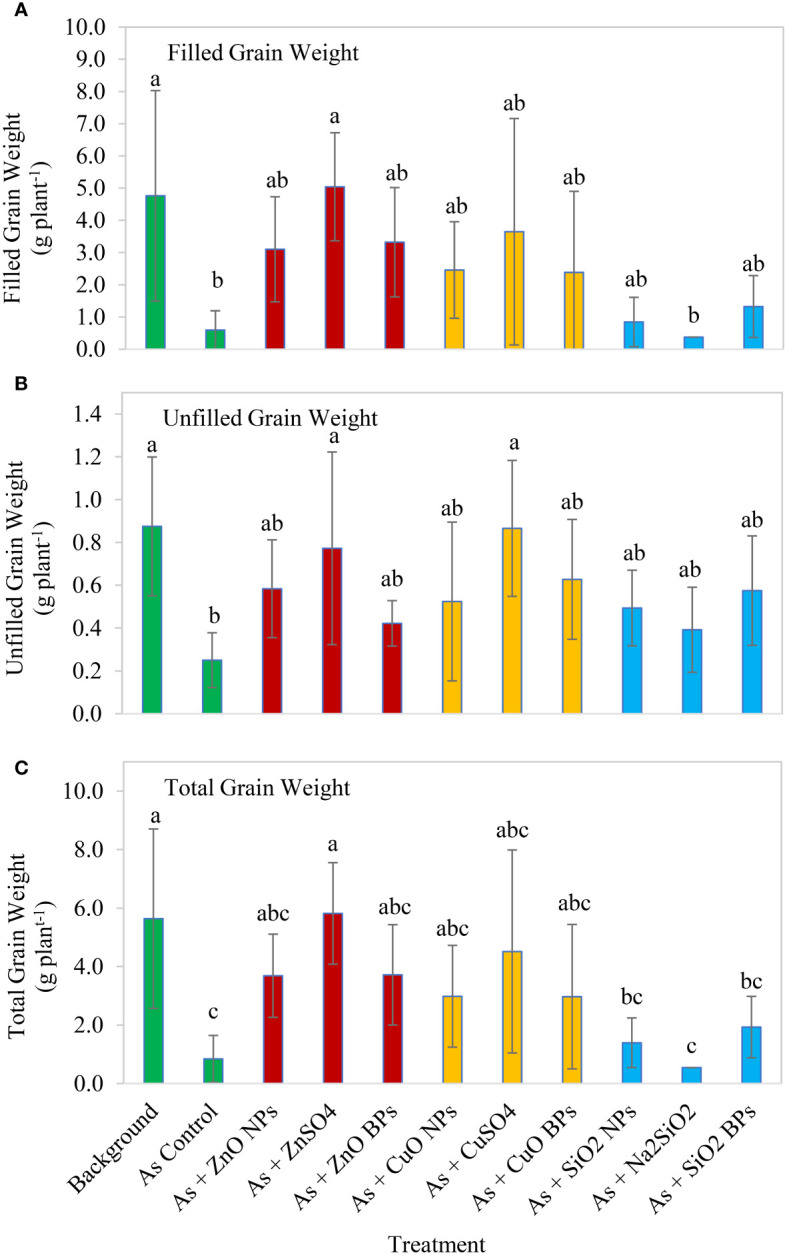
Effect of three metallic oxide nanoparticles (NPs), ionic counterparts, and bulk particles (BPs) on filled grain yield **(A)**, unfilled grain yield **(B)**, and total grain yield **(C)** of rice. Eleven treatments included: one background soil without amendment; one background soil with arsenic addition only (As control); background soil with As addition and amendment of a metallic oxide nanoparticle (ZnO NPs, CuO NPs, or SiO_2_ NPs); background soil with As addition and amendment of a ionic counterpart (ZnSO_4_, CuSO_4_, or Na_2_SiO_2_); and background soil with As addition and amendment of a bulk particle (ZnO BPs, CuO BPs, or SiO_2_ BPs). Letters on the bars denote statistical significance.

Filled grain weight, unfilled grain weight, and the total grain dry weight exhibited significant correlations (r values ≥ 0.28, *p* values ≤ 0.05) and shared a similar trend (**Table 2**; **Figures 2A-C**). As contamination significantly (*p* values ≤ 0.05) reduced the filled grain, unfilled grain, and total grain dry weight by 87.5%, 66.7%, and 85.7%, respectively, compared with the background control. Interestingly, in soils contaminated by As, application of ZnSO_4_ or CuSO_4_ significantly recovered the filled grain weight, unfilled grain weight, and total grain weight to various degrees. Among the three fertilizer forms, the ionic form of Zn and Cu resulted in the highest filled grain weight, unfilled grain weight, and total grain weight; however, the ionic form of Si resulted the lowest filled grain weight, unfilled grain weight, and total grain weight.

**Table 2:** Pearson’s correlations (*r*) and *p* value between parameters.

	Panicle Number		Plant Height		Shoot Biomass		Panicle Dry Wt		Unfilled and Filled Grain Wt		Filled Grain Wt		Unfilled Grain Wt		1000-Unfilled Grain Wt		1000-Filled Grain Wt		Unfilled Grain Number		Filled Grain Number		Filled/Unfilled Ratio		Grain As Conc		Husk As Conc		Total As Conc	
SPAD	-0.14		-0.20		0.22	*	-0.23	*	-0.21		-0.20		-0.13		0.13		-0.11		-0.09		-0.15		0.00		-0.30		-0.14		-0.21	
Panicle Number			0.29	**	0.20		0.55	***	0.49	***	0.41	***	0.70	***	-0.02		-0.02		0.67	***	0.37	**	-0.08		0.05		0.02		0.04	
Plant Height					0.17		0.42	***	0.33	**	0.29	*	0.31	**	0.17		0.32	**	0.20		0.21		0.17		-0.36	*	-0.14		-0.24	
Shoot Dry Biomass							0.06		0.08		0.03		0.35	**	0.01		-0.07		0.38	***	0.15		-0.12		-0.23		-0.04		-0.12	
Panicle Dry Wt									1.00	***	0.99	***	0.31	**	0.05		0.44	***	0.20		0.99	***	0.59	***	-0.35		-0.34		-0.37	*
Unfilled and Filled Grain Wt											0.99	***	0.28	*	0.01		0.45	***	0.18		0.99	***	0.60	***	-0.36	*	-0.35		-0.37	*
Filled Grain Wt													0.15		0.01		0.47	***	0.06		0.99	***	0.66	***	-0.35		-0.35		-0.37	*
Unfilled Grain Wt															-0.03		-0.15		0.96	***	0.11		-0.34	**	-0.13		-0.05		-0.08	
1000-Unfilled Grain Wt																	0.25	*	-0.18		-0.03		0.61	***	-0.01		0.02		0.01	
1000-Filled Grain Wt																			-0.17		0.40	***	0.57	***	-0.11		-0.17		-0.16	
Unfilled Grain Number																					0.05		-0.46	***	-0.04		0.09		0.04	
Filled Grain Number																							0.62	***	-0.35		-0.32		-0.36	
Filled/Unfilled Ratio																									-0.15		-0.18		-0.18	
Grain As Conc																											0.74	***	0.89	***
Husk As Conc																													0.96	***

*, significant at the 0.05 probability level; **, significant at the 0.01 probability level; ***, significant at the 0.001 probability level. SPAD denotes the chlorophyll SPAD index; Panicle Number indicates the number of panicles per plant (panicles plant^-1^); Plant Height is measured in centimeters (cm); Shoot Dry Biomass refers to the dry weight of shoot biomass per plant (g plant^-1^); Panicle Dry Wt represents the dry weight of panicles per plant (g plant^-1^); Unfilled and Filled Grain Wt denotes the weight of unfilled and filled grains per plant (g plant^-1^); Filled Grain Wt indicates the weight of filled grains per plant (g plant^-1^); Unfilled Grain Wt refers to the weight of unfilled grains per plant (g plant^-1^); 1000-Unfilled Grain Wt represents the weight of 1000 unfilled grains (g); 1000-Filled Grain Wt represents the weight of 1000 filled grains (g); Unfilled Grain Number denotes the number of unfilled grains per plant; Filled Grain Number indicates the number of filled grains per plant; Filled/Unfilled Ratio stands for the ratio of the number of filled grains to the number of unfilled grains; Grain As Conc represents the concentration of As in the hulled grain (mg kg^-1^ dry weight); Husk As Conc indicates the concentration of As in the grain husk (mg kg^-1^ dry weight).

**Figure 3:**
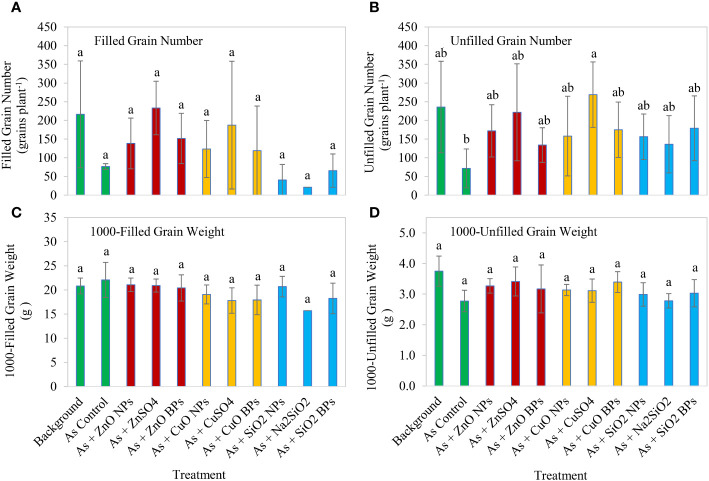
Effect of three metallic oxide nanoparticles (NPs), ionic counterparts, and bulk particles (BPs) on filled grain number per plant **(A)**, unfilled grain number per plant **(B)**, 1000-filled grain weight **(C)**, and 1000-unfilled grain weight **(D)** of rice. Eleven treatments included: one background soil without amendment; one background soil with arsenic addition only (As control); background soil with As addition and amendment of a metallic oxide nanoparticle (ZnO NPs, CuO NPs, or SiO_2_ NPs); background soil with As addition and amendment of a ionic counterpart (ZnSO_4_, CuSO_4_, or Na_2_SiO_2_); and background soil with As addition and amendment of a bulk particle (ZnO BPs, CuO BPs, or SiO_2_ BPs). Letters on the bars denote statistical significance.

For As contaminated treatments, application of ZnO, CuO, or SiO_2_ nanoparticles did not affect rice yield components, including the filled grain number, 1000-filled grain weight, or 1000-unfilled grain weight (**Figures 3A-D**). In contrast, As contamination significantly reduced the unfilled grain number compared to the CuSO_4_ treatment.

### 3.3 As accumulation and toxicity in rice

**Figure 4:**
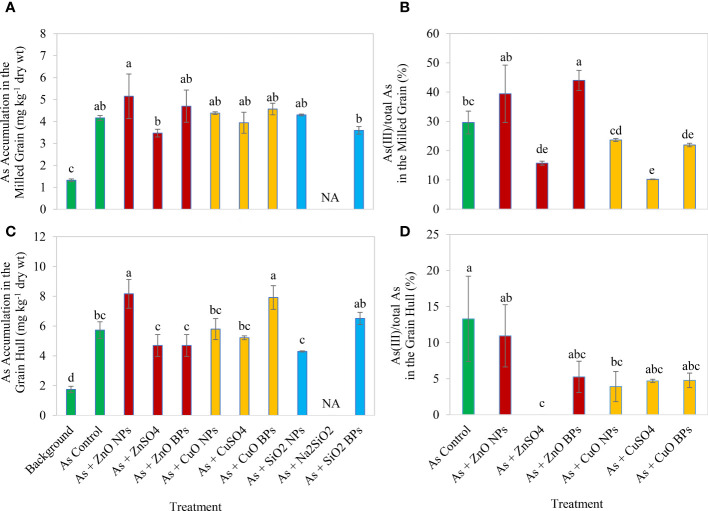
The total arsenic (As) accumulation in the milled rice grains **(A)** and grain hulls **(B)** and the percentage of As(III) of the total As in the milled rice grains **(C)** and grain hulls **(D)**. Milled grains are rice grains with the outer shell being removed, and the hulls are outer shell of rice grains. Eleven treatments included: one background soil without amendment; one background soil with arsenic addition only (As control); background soil with As addition and amendment of a metallic oxide nanoparticle (ZnO NPs, CuO NPs, or SiO_2_ NPs); background soil with As addition and amendment of a ionic counterpart (ZnSO_4_, CuSO_4_, or Na_2_SiO_2_); and background soil with As addition and amendment of a bulk particle (ZnO BPs, CuO BPs, or SiO_2_ BPs). Letters on the bars denote statistical significance.

The presence of 5 mg kg^-1^ arsenic significantly (*p* ≤ 0.05) increased the accumulation of As in both milled rice grains and hulls when compared to the background soil. Specifically, the total As accumulation increased by 3.5 times in the milled grains and 3.3 times in the hulls (**Figures 4A, B**).

Among the Zn treatments, the ZnSO_4_ amendment considerably reduced the accumulation of total arsenic in the grains and the As^III^/total As ratio. Specifically, the As accumulation in the grains was reduced by 17%, and the As^III^/total As ratio was reduced by 47%.

All three forms of Cu fertilizers significantly reduced grain As^III^/total As ratio in the milled grain and the grain hull, but none of the Cu fertilizers reduced the As accumulation (**Figures 4C, D**). Compared to the As control treatment, adding CuO NPs, CuSO_4_, and CuO BPs significantly reduced the As^III^/total As ratio in the milled grain by 20.2%, 65.6%, and 26.0%, respectively; and significantly reduced the As^III/^total As ratio in the grain hull by 70.6%, 64.7%, and 64.1%, respectively. Among the three types of Cu fertilizers, CuSO_4_ fertilizer was the most effective in reducing As^III^/total As ratio in the milled grain, while CuO NPs fertilizer was the most effective in reducing As^III^/total As ratio in the grain hull.

## 4 Discussion

### 4.1 Comparison of ZnO, CuO, and SiO_2_ nanoparticles, ionic counterparts, and bulk particles on rice plant growth under arsenic contamination

In this study, we observed that the presence of As contamination had a significant impact on several agronomic parameters of rice, including plant canopy height and panicle number. This reduction in plant height is consistent with the findings of Shaibur et al. (2006), who reported an 11% decrease in rice shoot length when exposed to 6.7 µmol L^−1^ As in a hydroponic setting. Farooq et al. (2016) also supported these observations by suggesting that high soil As contamination can induce oxidative stress, damage plant cell membranes, and ultimately lead to reduced photosynthesis and growth. Chlorophyll is a critical pigment for plant photosynthesis, and its content plays a significant role in determining plant growth rates. In our study, the addition of 5 mg kg^-1^ As did not significantly affect the chlorophyll index, as indicated by the SPAD value. However, Rahman et al. (2007) found a substantial reduction in chlorophyll-a and -b contents in rice varieties when exposed to higher As concentrations ranging from 10 to 30 mg kg^-1^. Similarly, Gaikwad et al. (2020) noted a reduction in chlorophyll content of rice plants when As concentrations ranged from 1.2 to 2.6 mg kg^-1^. These findings suggest that the effect of As on chlorophyll content in rice plants may be dose-dependent, with limited impact at lower As levels, such as 5 mg kg^-1^, and more pronounced effects as the As concentration increases. Further research with a wider range of arsenic concentrations is needed to fully understand the threshold values of chlorophyll sensitivity to As stress. Furthermore, our study found that the presence of 5 mg kg^-1^ As did impact rice shoot dry biomass. This observation in Hockley silt loam, characterized by 19% silt and 15% clay content, and possessing 8520 mg kg^-1^ total C, stands in contrast to findings from hydroponic experiments conducted by Abedin et al. (2002) and Yan et al. (2021). These studies reported a considerable reduction in rice straw biomass with when As concentration was introduced. The variations indicate that the sensitivity of rice plants to arsenic stress is intricately linked to environmental factors. The role of soil characteristics in mitigating As adverse impact on plant growth cannot be overstated. Soils rich in clay or organic matter content tend to effectively retain As, thereby limiting its accessibility to rice plants (Bakhat et al., 2019). Overall, our findings emphasize the importance of considering the dose of As, soil properties, and environmental factors when evaluating the influence of As on rice growth and development.

Under As stress, we found that CuO NPs, ZnO NPs, and SiO_2_ NPs had no discernible advantages over their ionic and bulk counterparts on rice growth parameters, including plants height, chlorophyll index, shoot dry biomass, and panicle numbers. By contrast, the ZnSO_4_ and CuSO_4_ amendments significantly increased the panicle number of rice under arsenic stress when compared to the As control treatment. Our findings are consistent with previous studies (Das et al., 2008; Xi et al., 2019). Das et al. (2008) observed that the extractable As concentration increased with decreasing ZnSO_4_ application. Xi et al. (2019) found that increasing CuSO_4_ concentrations from 0.2 to 1 g L^-1^ in wastewater treatment led to a higher As removal rate. Our results align with these observations and further support the notion that ZnSO_4_ and CuSO_4_ amendments have a positive influence on rice growth under As stress.

However, our study also revealed a noteworthy contrast in the case of ZnO and CuO nanoparticles. The addition of these nanoparticles at a concentration of 100 mg kg^-1^ led to a significant (*p* ≤ 0.05) reduction in the chlorophyll SPAD index in rice leaves compared to the As control treatment. This decrease in chlorophyll levels suggests a potential inhibition of ZnO and CuO nanoparticles to rice photosynthesis and prompts further consideration about their broader applications in agriculture. The adverse effects of ZnO and CuO nanoparticles on chlorophyll levels can be attributed to their small size and increased surface area, which may facilitate the generation of reactive oxygen species (ROS) and induce oxidative stress and damage to cellular structures, including chloroplasts where chlorophyll is primarily located. Nevertheless, it’s important to note that Yan et al. (2021) reported positive effects when lower levels of ZnO NPs (10–100 mg L^–1^) were added, leading to increased rice resistance to As toxicity and higher rice shoot biomass. The inconsistent results suggest the complexity of nanoparticle-plant interactions and emphasize the influence of various factors, including nanoparticle concentration, exposure duration, and rice varieties. To gain a comprehensive understanding of the threshold values and specific mechanisms that underlie the varying impacts of different nanoparticles on rice growth under As stress, further research is warranted. This includes investigations into the precise mechanisms responsible for nanoparticle-induced toxicity and their potential mitigation strategies. Additionally, research exploring the influence of different nanoparticle sizes and surface coatings on rice plants is essential to inform safe and effective applications of nanotechnology in agriculture. Overall, our study contributes to the understanding of the intricate interactions between nanomaterials and plants, shedding light on the potential benefits and risks of nanotechnology in agricultural contexts.

### 4.2 Comparison of ZnO, CuO, and SiO_2_ nanoparticles, ionic counterparts, and bulk particles on rice grain yield under arsenic contamination

Our study revealed a significant reduction in filled rice grain yield in the presence of As contamination, which aligns with previous research (Abedin et al., 2002; Abbas et al., 2018). This reduction in grain yield is likely related to the adverse effects of As on photosynthesis and nutrient uptake. Arsenic toxicity can lead to chlorophyll degradation, hindering photosynthesis, and disrupts nutrient absorption, limiting essential nutrients for grain development. Our findings underscore the need for research to explore and develop effective strategies to counteract the adverse effects of As on rice plants, including the use of soil amendments.

Regarding soil amendments, in this study, we found that the effectiveness of different forms of fertilizer in recovering grain yield under As contamination varied for the three micronutrients. For Zn and Cu treatments, filled grain weight, unfilled grain weight, and total grain weight were highest in soils treated with ionic counterparts (ZnSO_4_ or CuSO_4_) as compared to the other two forms. However, the trend reversed for Si treatments. Furthermore, we found that NPs did not confer advantages over their ionic counterparts and bulk particles in improving rice grain yield under As contamination. Previous research has reported both positive and negative effects of NPs, such as ZnO NPs, CuO NPs, and SiO_2_ NPs, on seed germination, plant growth, and disease suppression (Wei et al., 2021). Some studies have documented beneficial effects, including enhanced plant biomass, modifications in plant tissue differentiation, and activation of plant defense mechanisms (Dimkpa et al., 2017; Mittal et al., 2020). The positive effects of ZnO NPs, CuO NPs, and SiO_2_ NPs include increasing plant biomass and physiology, modifying plant tissue differentiation, and activating plant defense systems (Mittal et al., 2020), which are owing to their active packing and bioactive ingredients delivery systems (Sabeena et al., 2022). The negative effects or safety concerns of ZnO NPs, CuO NPs, and SiO_2_ NPs are related to their potential phytotoxicity such as reducing germination rate, biomass, root, and shoot length, as well as their accumulation in the soil and edible plant tissues and grains (Ahmed et al., 2018; Naz et al., 2020). Some of the reasons could be oxidative stress induction, cell death, DNA damage, increased activity of stress enzymes, and disruption of photosynthesis and transpiration rates (Rajput et al., 2018).

The diverse effects of NPs underscore the necessity for a more comprehensive understanding of their interactions with various plant species, environmental conditions, and specific contaminants, such as As. To harness the potential benefits of NPs and mitigate their potential risks, future research should aim to elucidate the intricate mechanisms governing the interactions between NPs and plants, as well as their responses to different stressors, including As contamination.

### 4.3 Comparison of ZnO, CuO, and SiO_2_ nanoparticles, ionic counterparts, and bulk particles on As accumulation and toxicity in rice under arsenic contamination

In our study, we observed a significant increase in the accumulation of As in both milled grains (3.5 times) and hulls (3.3 times) when rice was exposed to As contamination, in comparison to the background soil. This observation suggests the strong capacity of rice plants to absorb and accumulate significant amounts of As in their edible grains and protective hulls when grown in As-contaminated soil. Our finding also aligns with previous research (Rahman et al., 2007). Ma et al. (2008) delved further into the mechanisms underlying this phenomenon and demonstrated that As transport to rice grains and hulls is a complex process that involves various mechanisms, including root uptake through phosphate/silicon transporters, translocation via the xylem, and the deposition in grain and hull tissues. These findings underscore significant risks associated with rice cultivation in As-contaminated regions, particularly in terms of food safety and human health. To ensure the safety and sustainability of rice production in such areas, further research is imperative to explore strategies for reducing arsenic uptake by rice plants and developing low-As accumulation rice varieties.

Interestingly, our study revealed that under As contamination, the amendment of ZnSO_4_ led to a complete recovery of rice yield to the background level. This recovery was accompanied by a 17% reduction in grain As accumulation and a notable 47% decrease in the As^III^/total As ratio. The efficacy of ZnSO_4_ in reducing As accumulation is in line with Wong et al. (2019), who observed a significant interaction between zinc deficiency and As exposure. Our findings suggest that ZnSO_4_ amendment not only aids in restoring rice yield but also impacts the mechanisms governing As accumulation in rice grains. This implies the potential of ZnSO_4_ as an effective approach for mitigating the adverse effects of As contamination on rice. Furthermore, our study underscores the necessity for further research to elucidate the specific mechanisms that underlie the observed interactions between Zn and As in rice plants.

We also found that Cu amendment, regardless of the form used, contributed to a significant reduction in the grain As^III^/total As ratio, both in milled grains and grain hulls. The potential of Cu amendments to mitigate As accumulation in rice grains aligns with recent research by Wu et al. (2022), who reported that the addition of 100 mg kg^–1^ CuO NPs led to increased microbial diversity and enhanced gene abundance related to As cycling. This microbial activity, in turn, resulted in decreased As accumulation in grains. These findings shed light on the interactions between Cu, microbial communities, and As dynamics in rice fields. However, it is essential to exercise caution when considering Cu amendment, particularly if it surpasses the maximum Cu limit (1.3 ppm) in drinking water as set by the EPA’s National Primary Drinking Water Regulations (NPDWR), due to its potential negative environmental impact. Nevertheless, further research is necessary to determine the threshold of Cu amendment that effectively mitigates As accumulation while staying within EPA guidelines, ensuring both environmental sustainability and As risk mitigation in rice cultivation.

In addition, we observed limited impact of Si amendments on rice growth, grain yield, and As accumulation in this study. However, Li et al. (2009) found that the addition of Si fertilizer markedly decreased As accumulation in rice shoots and the Asi concentration in the rice grain in a greenhouse study. The inconsistent findings suggest that the efficacy of Si in mitigating As accumulation in rice may be context-dependent and underscore the importance of the soil conditions and geochemical factors in modulating the effects of Si amendments. Bogdan and Schenk (2008) reported that Si availability in soil has a substantial influence on As uptake by rice. And Ma et al. (2008) explained that this is because of the shared uptake pathway between Si and arsenite. In light of these variations, further research is essential to elucidate the specific mechanisms governing the role of Si in As uptake and translocation in rice plants under diverse environmental conditions.

## 5 Conclusions

In conclusion, the presence of As in soil significantly reduced the rice plant height, panicle number, and grain yield and increased the As accumulation in both milled rice grains and hulls. The results highlight the need for effective management strategies to mitigate the negative impact of As on rice plant growth and ensure food security. Under As contamination, the application of Zn and Cu amendments increased rice grain yield by 4.6–7.3 times and 3.8–5.6 times, respectively. Among the three micronutrient forms, the application of ZnSO_4_ and CuSO_4_ was the most effective for reducing As toxicity in rice plants under As contamination. Both ZnSO_4_ and CuSO_4_ amendments recovered grain yield to the background level, and they significantly reduced grain As^III^/total As ratio by 46.9% and 65.6%, respectively. The results revealed that Zn and Cu amendments, especially the salt form, could promote rice yield and lower the risk of As accumulation in rice grains under As contamination. However, further research is necessary to determine the threshold of Cu amendment that effectively mitigates As accumulation while staying within EPA guidelines. Importantly, we found that NPs do not have advantages over their ionic counterparts and bulk particles in promoting rice growth under As contamination, although certain NPs did reduce As toxicity. The results suggest nanotechnology should not be embraced indiscriminately even though it offers many promising benefits. However, this study was conducted in a condition-controlled and monitored greenhouse using one variety of rice. Although we designed the experiment to our best knowledge to compare the effectiveness of ZnO, CuO, and SiO_2_ nanoparticles, ionic counterparts, and bulk particles, the results could vary with different rice varieties, under different arsenic concentrations, in different soil types, and under different climate conditions. Future field trials would be needed to validate the conclusions drawn in this study to achieve a more extensive understanding of the effects of different nutrient amendments to rice in the presence of As contamination.

## Data availability statement

The original contributions presented in the study are included in the article/supplementary material. Further inquiries can be directed to the corresponding author.

## Author contributions

XL: Data curation, Formal analysis, Investigation, Methodology, Software, Validation, Visualization, Writing – original draft, Writing – review & editing. XW: Formal analysis, Investigation, Methodology, Validation, Writing – review & editing. XM: Conceptualization, Funding acquisition, Methodology, Resources, Supervision, Validation, Writing – review & editing. WS: Conceptualization, Data curation, Funding acquisition, Writing – review & editing. KC: Formal analysis, Software, Visualization, Writing – review & editing. FD: Conceptualization, Funding acquisition, Methodology, Project administration, Resources, Supervision, Writing – original draft, Writing – review & editing.
